# Rhinosinusitis and Stroke: A Systematic Review

**DOI:** 10.7759/cureus.40923

**Published:** 2023-06-25

**Authors:** Anna-Maria Papadopoulou, Athanasia Marinou

**Affiliations:** 1 Department of Otolaryngology, Head and Neck Surgery, "G.Gennimatas" General Hospital of Athens, Athens, GRC

**Keywords:** computed tomography, risk factor, sinusitis complication, magnetic resonance, atherosclerosis, ischemic stroke, chronic rhinosinusitis, acute rhinosinusitis

## Abstract

Rhinosinusitis is one of the most common inflammatory diseases. It has been recognized that intracranial vessels are involved and there might be an association with stroke occurrence. The aim of this study was to evaluate the association between rhinosinusitis and cardiovascular diseases, especially stroke, through a literature review. The review was conducted according to the Preferred Reporting Items for Systematic Reviews and Meta-Analysis (PRISMA) guidelines. We performed on PubMed a literature search from February 2000 to February 2022, using the search terms ‘rhinosinusitis’ OR ‘chronic rhinosinusitis’ AND ‘stroke’ OR ‘ischemic stroke’. Fourteen studies were eligible and included in the analysis. Overall, the studies encompassed a total of 1,006,338 patients included in this review. All studies concluded that there is a statistically significant correlation between clinical or radiological sinus inflammation and the risk of stroke, which is independent of traditional stroke risk factors. In conclusion, rhinosinusitis is associated with an increased incidence of stroke.

## Introduction and background

Rhinosinusitis is a worldwide prevalent upper respiratory inflammation of the mucosa of the nose and paranasal sinuses and is classified into acute (<12 weeks) and chronic (>12 weeks) [1]. Left untreated or undertreated, it can lead to several serious infectious and vascular complications related to the central nervous system, such as meningitis, epidural, or subdural empyema, brain abscess, cranial nerve palsies, mycotic aneurysms, intracranial hypertension, and cavernous or sagittal sinus thrombophlebitis [1,2]. In addition, a close relationship between chronic inflammatory diseases and cardio-cerebrovascular disease has been noticed [3,4]. Interestingly, previous studies have revealed an association between chronic rhinosinusitis (CRS) and a higher risk for acute myocardial infarction and stroke [5]. Stroke is one of the leading causes of morbidity and mortality worldwide. The exact relation between stroke and rhinosinusitis is still not fully elucidated. The predominant theory suggests that the anatomic proximity of the paranasal sinuses and internal carotid artery or brain could lead to the expansion of the inflammation of the sinuses to the intracranial vasculature. Other theories include inflammation-mediated emboli or spasms of cerebral arteries, adverse reactions to medicine, or complications following sinus surgery [6,7].

In the present study, our objective was to evaluate the association between rhinosinusitis and cardiovascular diseases, especially stroke, through a literature review.

## Review

Material and methods

This systematic review was performed in accordance with the PRISMA guidelines. Eligible articles were identified by a search of the PubMed bibliographical database for the period from September 2001 up to February 2022. The study protocol was agreed upon by both co-authors. The search strategy included the following keywords (“rhinosinusitis” OR “chronic rhinosinusitis”) AND (“stroke” OR “ischemic stroke”). Language restrictions were applied (only articles in English were considered eligible); two investigators (AMP and AM), working independently, searched the literature and extracted data from each eligible study. Reviews were not eligible, while all prospective and retrospective studies, as well as case reports, were eligible for this systematic review. Manuscripts that did not state the names of the authors were excluded. In addition, we checked all the references of relevant reviews and eligible articles that our search retrieved, so as to identify potentially eligible conference abstracts. Titles of interest were further reviewed by abstract. Finally, reference lists of eligible studies were manually assessed in order to detect any potentially relevant article (“snowball" procedure).

Results

Article Selection and Study Demographics

**Figure 1:**
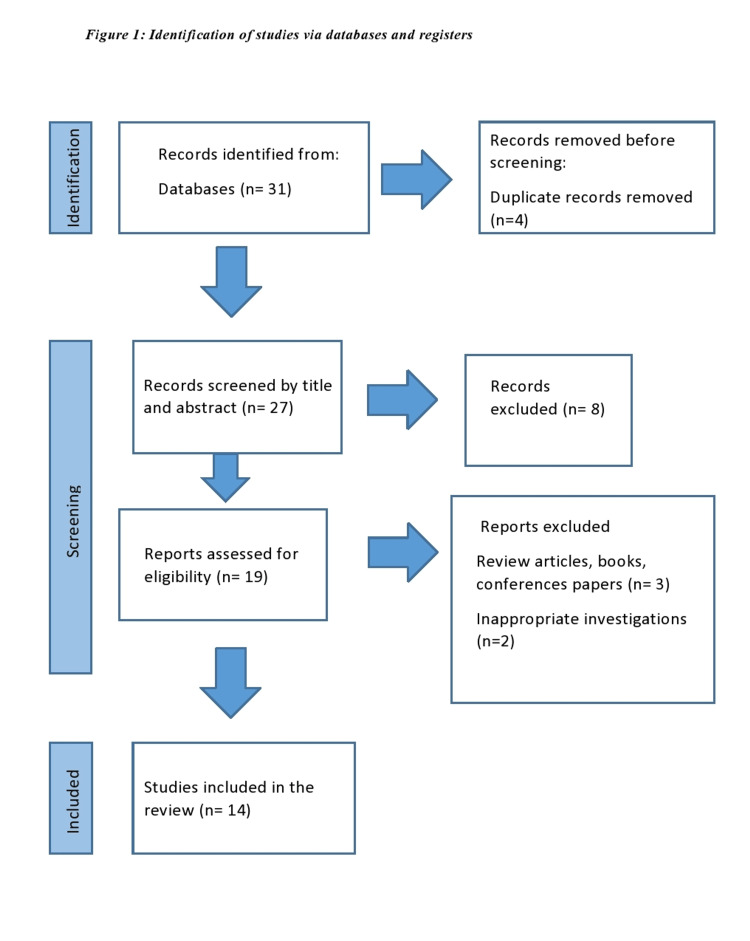
Flowchart of the search strategy

Following the screening of titles and abstracts, the search strategy retrieved 27 articles that were evaluated for full-text evaluation. Fourteen studies were deemed eligible and were included in the analytic cohort. Overall, these studies encompass a total of 1,006,338 patients that have been included in this systematic review. The search strategy is depicted in Figure 1.

Main Findings

Table 1 summarizes findings from the articles reporting a correlation between rhinosinusitis and stroke.

**Table 1:** Correlation between rhinosinusitis and stroke CRS: chronic rhinosinusitis, HR: hazard ratio, L-M: Lund-Mackay

Author	Year	Type of Study	n	Results
Wu et al [1]	2012	Prospective cohort study	268277; Patients: 53656; Controls: 214624	(1) Patients with rhinosinusitis were more likely to suffer strokes than controls (adjusted HR, 1.39; 95% CI, 1.28-1.50; (2) The HR of stroke was 1.39 (95% CI, 1.28-1.51) for ARS patients, and 1.34 (95% CI, 1.04-1,74) for CRS patients.
Kang et al. [2]	2013	Population-based cohort study	63384; Patients: 15846; Controls: 47538	subjects with CRS were more likely than comparison subjects to have a diagnosis of ischemic stroke during the 5-year follow-up period (HR 1⁄4 1.34, 95% CI 1⁄4 1.18–1.53)
Lee et al. [3]	2018	National cohort study	114795; Patients: 22959; Controls: 91836	Significantly increased HR for hemorrhagic and ischemic stroke in the CRS patients compared to controls (adjusted HR = 2.43, 95% confidence interval [CI] = 2.10– 2.80 for hemorrhagic stroke; adjusted HR = 1.76, 95% CI = 1.61–1.92 for ischemic stroke)
Stryjewska‐Makuch et al. [4]	2021	Retrospective case-control study	238 Patients: 163; Controls: 75	Inflammatory sinus lesions of moderate or high severity were more often observed in patients with stroke than in the control group and they mainly involved the ethmoid sinuses.
Perez Barreto et al. [6]	2000	Case report	4	Large vessel stroke involving the internal carotid artery territory in patients with extensive disease of the sphenoid and other sinuses
Righini et al. [7]	2009	Case report	1	A 28-year-old woman with acute sphenoid sinusitis complicated by ischemic stroke in the left caudate nucleus, lentiform nucleus, and posterior part of the internal capsule.
Rochat et al. [8]	2001	Case report	1	A 10-year-old girl with severe acute pansinusitis and ischemic stroke in the right lentiform nucleus and the anterior part of the right internal capsule
Fabre et al. [9]	2018	Case report	1	middle cerebral artery ischemic stroke secondary to inflammatory arteritis of the left internal carotid artery in a context of bilateral acute maxillary sinusitis in a 6-year-old girl.
Davison et al. [10]	2021	Case report	1	acute stroke in the setting of severe acute pansinusitis in a 9-year-old male
Fu et al. [11]	2015	Case report	1	Basilar artery territory stroke secondary to invasive fungal sphenoid sinusitis in a 38-year-old man
Jeon et al. [12]	2022	Longitudinal follow-up study	32760; Patients: 6552; Controls: 26208	(1) Significantly increased risk for stroke in CRS patients compared to controls; (2) Significantly higher HR of stroke in the absence of nasal polyps than in the presence of nasal polyps
Kim et al. [13]	2019	Population-based, long-term longitudinal study	44286; Patients: 14762; Controls: 29524	HR of stroke in patients with CRS versus controls: 1.16 (95% CI, 1.08-1.24)
Rosenthal et al. [14]	2016	Retrospective study	173	Incidental paranasal sinusitis (MRI) was strongly associated with cerebrovascular disease (p<0,001)
Puz et al. [15]	2021	Retrospective study	311	Significant difference in the neurological status of stroke patients between mild and severe L-M score (p = 0.02)

Six case reports which included nine patients were selected for this systematic review [6-11]. The majority of them presented cases of ischemic stroke as a complication of acute rhinosinusitis. Four cases (44%) concerned young children [6,8-10] and the rest five cases (56%) concerned adults, with patient ages ranging from 28 to 62 years [6,7,11]. Two patients (22%) suffered from diabetes, which is thought not only to predispose to paranasal sinus infection but also to be responsible for the asymptomatic nature of this infection and consequent late diagnosis [9,11]. The sphenoid sinus was involved in almost all patients (89%). One patient (11%) was diagnosed with acute pansinusitis [8], three patients (33%) had acute isolated sphenoid sinusitis [6,7,11], while Fabre et al. were the first to report a case of stroke secondary to acute maxillary sinusitis [9]. According to the magnetic resonance images, which confirmed the ischemic strokes, most of them were secondary to narrowing or occlusion of the internal carotid artery adjacent to involved paranasal sinuses, suggesting inflammatory arteritis [6,7,9]. Only Davison et al. described a case of acute sinusitis presenting as stroke with ischemic changes in MR imaging, however, without any evidence of thrombosis in the major vessels on MRI, MRA, or CT [10]. None of the patients demonstrated venous pathology. All patients were successfully treated by endoscopic surgery and broad-spectrum antibiotics, with the exception of a diabetic man with invasive mucormycosis that led to a basilar artery territory stroke and subsequent death [11].

The rest of the collected studies were cohort studies that investigated the association between rhinosinusitis and the incidence of ischemic or hemorrhagic stroke. Although different methodological approaches were followed, all of them concluded that CRS can be considered a relevant risk factor for stroke. In greater detail, a recent longitudinal follow-up study proved that CRS patients had a significantly higher prevalence of stroke compared to controls, even after adjusting for traditional cardiovascular risk factors, which also is in agreement with the results of Lee et al [3,12]. Notably, the hazard ratio (HR) of stroke was significantly higher in patients without nasal polyps than in those with nasal polyps [12]. Wu et al. also compared the risk of stroke between CRS patients and controls and found that patients had a 1.39-fold increased risk during a three-year follow-up period [1]. The HR remained significantly higher after adjusting for demographic characteristics and medical comorbidities [1]. Significantly increased HR of stroke in the CRS group compared to controls, regardless of sociodemographic factors was also observed in two other studies (HR:1.16 and 1.33 respectively) [2,13]. These results are consistent with another large national cohort study with a 10-year follow-up period which, interestingly, highlights that the occurrence of stroke increased in the course of time following CRS, especially within one year [3]. Based on this finding, the authors pointed out the significance of early and aggressive CRS treatment in the prevention of stroke and its complications [3]. Moreover, the same study reported an increased risk of both ischemic and hemorrhagic stroke in CRS patients [3], whereas Kang et al. observed a statistically increased risk of ischemic stroke only in patients with CRS [2]. This disparity could be explained due to different follow-up periods, since the slow and gradual evolution of CRS may demand a longer time for intracranial complications to occur [3]. Furthermore, the number of intracranial hemorrhage cases is quite small, which could create an artifact of inadequate statistical power [2].

The remaining studies investigated the association between incidental paranasal sinusitis and cerebrovascular disease. For example, a strong association was proved by Rosenthal et al., who studied a random sample of magnetic resonance (MR) brain scans (p<0,001) [14]. Another case-control study demonstrated that patients with ischemic stroke who underwent thrombectomy presented chronic inflammation of the paranasal sinuses with the same rate as atrial fibrillation and more frequently than diabetes and smoking [4]. More specifically, lesions of moderate or high severity which mainly involved the ostiomeatal complex, ethmoid, frontal and maxillary sinuses, were more frequently encountered in stroke patients than in controls [4]. In addition, older patients with stroke had larger lesions. On the contrary, Lee et al. showed that CRS patients under 60 years of age were at greater risk of stroke [3]. Moreover, the impact of CRS on the short-term prognosis in stroke patients has also been confirmed [15]. The neurological status in patients with mild signs of CRS, as evaluated with CT scans, was significantly better than in those with moderate or severe lesions. As a result, the authors proposed the CT features of CRS as an early prognostic tool for patients with ischemic stroke. Notably, when moderate or severe CRS is diagnosed, intensive maximal medical therapy or interventional treatment should be started as early as possible, as this may improve the short-term efficacy of endovascular treatment of patients with stroke [15].

Furthermore, it can be implied by the literature that there is an association between ARS and stroke based on the findings by Wu et al. [1] and the case reports reviewed in this article.

Finally, two studies demonstrated that CRS patients had a significantly higher prevalence of ischemic heart disease as well [12,13].

Discussion

Despite recent improvements in antibiotic therapies and surgical interventions, intracranial complications of rhinosinusitis can lead to disabilities in 25% of cases and even death in 10% [16,17]. All evidence supports that stroke may be considered a serious complication too, according to our literature review. The exact mechanism which explains the relation between rhinosinusitis and stroke is likely to be heterogeneous and has not been fully understood yet. Several possible explanations have been implicated to contribute to this association.

Firstly, some cases of sinusitis-related stroke were subsequent complications of intracranial infection, which can lead to cerebral vasculitis and brain blood flow disruption through direct spread into intracranial vessels [18]. Meningitis, for example, has been associated with cerebral ischemia, probably through vasospasm caused by inflammatory cytokines [19]. The association between acute infection and stroke is not dependent on particular microbial agents but it is a result of the inflammatory response to infection, which induces a procoagulant state [18].

Secondly, there is close anatomical proximity between the sinuses and the intracranial cavity, which are separated from each other by a thin bony wall [6,20]. In fact, the internal carotid artery lies immediately adjacent to the approximately 0.1-mm thin lateral wall of the sphenoid sinus [6,21]. Cases of sphenoid sinusitis complicated with carotid and basilar arteries occlusion have been described in the literature [1,6]. For example, Perez Barreto et al. presented a case of internal carotid artery stroke in a patient with extensive disease of the sphenoid sinus [6]. Bony dehiscence and true prolapse of the internal carotid artery into not only the sphenoid sinus but also into the posterior ethmoid cells are common, inducing direct contact of sinus mucosa with the artery [6]. Moreover, ethmoid cells, which are lined with a large mucosal surface relative to their volume, are often vascularized by anterior and posterior ethmoid arteries with the absence of bone canals, facilitating direct contact of the mucosa with the arteries [4]. Contiguous inflammation of the carotid artery canal through infratemporal (pericarotid) venous thrombosis extending to the cavernous sinus due to maxillary sinusitis has also been described [9].

The anatomical proximity and direct contact of the paranasal sinus mucosa with vessels can lead to perivascular inflammatory reactions [1], which can be of either infectious or non-infectious etiology [2]. For example, cases of septic thrombophlebitis of the internal jugular vein that lead to cerebral infarction in CRS patients have been reported (Lemierre’s syndrome) [22]. Intracranial hemorrhage or cerebral infarction can also be caused by infectious aneurysms [23]. On the other hand, high local concentrations of proinflammatory cytokines have been found in retained sinus fluids and sinus mucosa of patients with sinusitis [24,25]. Inflammation is thought to play a key role in atherosclerotic initiation, plaque rupture, thrombosis, and consequently stroke [1]. It has been demonstrated by many authors that inflammation adversely affects the integrity and function of endothelial cells [18]. Several inflammatory cytokines, such as interleukin-1, interleukin-6, interleukin-17, or C-reactive protein are responsible for immune and subendothelial smooth muscle cell activation, accelerating atherogenesis [18], thereby, leading to premature atherosclerosis with reduced cerebral flow and nerve tissue perfusion [26]. These inflammatory cytokines may also cross-activate the coagulation pathway by triggering the thrombin coagulation system and upregulating the fibrinolytic inhibiting protein, which leads to a higher chance of thrombus formation and thromboembolic events. In particular, interleukin-1 may result in perivascular inflammation and progression of internal carotid artery thrombosis, while interleukin-17, particularly in combination with tumor necrosis factor α, is thought to have a proinflammatory, procoagulant, and prothrombotic effect on blood vessels [1,18]. Besides, bacterial antigens and activated leukocytes can directly activate platelets [27]. Lipopolysaccharides of Gram-negative bacteria, which are very common in older adults with sinusitis, have been reported to induce stroke in rats [28]. In addition, inflammation of vessel walls can result in aneurysms and wall rupture accompanied by local hemorrhage [28].

The fact that sinusitis and cardiovascular diseases share some common predisposing factors could also explain their association. For instance, smoking is a risk factor for both conditions. It disturbs the mucociliary clearance of the sinuses but also contributes to a stroke mortality rate of approximately 15%, with a dose-response relationship [29]. Only three cohort studies adjusted their results for tobacco use [2,12,14]. There are other comorbidities of sinusitis that serve as nontraditional cardiovascular risk factors as well, such as allergy or asthma [2]. These conditions, except for being intimately associated with CRS, have been reported to increase the risk of stroke [2]. Other comorbidities that are suggested to be related to both CRS and stroke include gastroesophageal reflux and sleep difficulties [2]. Furthermore, sinusitis is thought to serve as a marker of dental and respiratory tract infections, which are frequently encountered in patients with acute ischemic stroke [6]. As a result, the association between sinusitis and stroke could be partly explained as a function of shared predisposing and confounding factors.

Finally, the treatment modalities of sinusitis may sometimes increase the hazard of stroke occurrence. Decongestant drugs, such as sympathomimetics, increase blood pressure and heart rate, which are predominant risk factors for ischemic stroke [30,31]. Decongestants have also been suggested to be related to intracranial hemorrhage [2]. Particularly, a study with a Korean population found that there was a 0.6% to 1.6% exposure rate of phenylpropanolamine which has been connected with hemorrhagic stroke [32]. Likewise, a case report described an arteriovenous malformation rupture that led to intracranial and subarachnoid hemorrhage in a decongestant user [33]. In addition, subarachnoid hemorrhage is a rare but disturbing sequela of endoscopic sinus surgery [2].

In nearly half of the collected cohort studies the CRS diagnosis was based on inflammatory lesions apparent on sinus imaging, either with CT or MRI scans [4,14,15]. The lack of data on the history of sinus complaints, severity, disease status, and possible treatment in accordance with the EPOS 2020 guidelines [34] limits these studies. Moreover, the accuracy of imaging modalities, especially MRI for sinonasal disease, is questionable. Lastly, most studies included in this review were case reports and retrospective cohorts. Therefore, the underlying mechanisms which can better explain the association between sinusitis and stroke could not be directly examined and analyzed.

## Conclusions

The present systematic review investigated the interesting and lesser-known association between sinusitis and stroke. Our results suggest that clinicians should be aware of the increased risk of stroke when dealing with patients with acute or chronic rhinosinusitis. Several potential explanations have been proposed for this relation, but larger prospective studies and trials are required to figure out further issues, such as whether this relationship is really causal, the pathomechanism of this association, how the severity of the disease contributes to the risk of stroke, which preventive strategies can be developed, and if there is an impact of sinusitis on the progression and therapy of the acute phase of stroke.
